# Progression of Cardio-Metabolic Risk Factors in Subjects Born Small and Large for Gestational Age

**DOI:** 10.1371/journal.pone.0104278

**Published:** 2014-08-12

**Authors:** Valentina Chiavaroli, Maria Loredana Marcovecchio, Tommaso de Giorgis, Laura Diesse, Francesco Chiarelli, Angelika Mohn

**Affiliations:** 1 Department of Paediatrics, University of Chieti, Chieti, Italy; 2 Center of Excellence on Aging, “G. d'Annunzio” University Foundation, University of Chieti, Chieti, Italy; University of Catanzaro Magna Graecia, Italy

## Abstract

**Background:**

Subjects born small (SGA) and large (LGA) for gestational age have an increased risk of cardio-metabolic alterations already during prepuberty. Nevertheless, the progression of their cardio-metabolic profile from childhood to adolescence has not been fully explored. Our aim was to assess potential changes in the cardio-metabolic profile from childhood to adolescence in subjects born SGA and LGA compared to those born appropriate (AGA) for gestational age.

**Methods:**

This longitudinal study included 35 AGA, 24 SGA and 31 LGA subjects evaluated during childhood (mean age (±SD) 8.4±1.4 yr) and then re-assessed during adolescence (mean age 13.3±1.8 yr). BMI, blood pressure, insulin resistance (fasting insulin, HOMA-IR) and lipids were assessed. A cardio-metabolic risk z-score was applied and this consisted in calculating the sum of sex-specific z-scores for BMI, blood pressure, HOMA-IR, triglycerides and triglycerides:high-density lipoprotein cholesterol ratio.

**Results:**

Fasting insulin and HOMA-IR were higher in SGA and LGA than AGA subjects both during childhood (all *P*<0.01) and adolescence (all *P*<0.01). Similarly, the clustered cardio-metabolic risk score was higher in SGA and LGA than AGA children (both *P*<0.05), and these differences among groups increased during adolescence (both *P*<0.05). Of note, a progression of the clustered cardio-metabolic risk score was observed from childhood to adolescence within SGA and within LGA subjects (both *P*<0.05).

**Conclusions:**

SGA and LGA subjects showed an adverse cardio-metabolic profile during childhood when compared to AGA peers, with a worsening of this profile during adolescence. These findings indicate an overtime progression of insulin resistance and overall estimated cardiovascular risk from childhood to adolescence in SGA and LGA populations.

## Introduction

Birth weight is now recognized to have important implications for cardio-metabolic health in adulthood. Children born small (SGA) and large (LGA) for gestational age are at greater risk of developing type 2 diabetes and cardiovascular disease as they age [1]–[6]. These groups have been shown to have metabolic alterations in childhood and adolescence, suggesting a phenotype of metabolic dysfunction in early life, finally leading to the emergence of disease in adulthood [2], [6]–[10].

The determinants of birth weight are multifactorial including genetics, ethnicity, maternal nutrition, obesity, smoking and diabetes [11], [12]. Birth weight is a marker of intrauterine nutrition, hence it is likely that the mechanisms mediating the increased metabolic risk of those born SGA are different to those born LGA. Nevertheless, decreased insulin sensitivity has a key role linking birth weight to the development of chronic disease in both these groups [6], [7]. Metabolic risk is further modulated by the post-natal growth pattern, and infants born SGA who show early catch-up growth are at increased risk [13]–[15]. Reduced insulin sensitivity and increased fat accumulation have also been detected in LGA subjects [15]–[17].

Most studies investigating the relationship between birth weight and metabolic risk have been cross-sectional [13]–[15], [17]. It is important to reassess cohorts of children prospectively to investigate whether markers of metabolic risk are stable or progress overtime.

We have previously shown in a cohort of prepubertal children that those born SGA or LGA were more insulin resistant and had greater oxidative stress than those born appropriate (AGA) for gestational age [15]. Further, these factors were greater in those who were obese.

Therefore, in the present study we have reassessed a group of adolescents born AGA, SGA and LGA, most of whom belonged to the previous cohort of prepubertal children, to describe how markers of metabolic risk change after puberty.

## Materials and Methods

### Ethics Statement

This study was approved by the Ethical Committee of the University of Chieti. Written informed parental consent, oral assent from children and written consent from adolescents were obtained.

### Recruitment

We recruited 115 Caucasian children, living in the district of Chieti (Abruzzo, Central Italy). These children were admitted to the Department of Pediatrics of the University of Chieti, for minor illness or cranio-facial trauma. During hospitalization, parents were informed about the study and children were invited to participate.

Children were eligible for the study if they were born at term (37–41 weeks of gestation) from singleton pregnancies. Children were excluded if they had a first degree relative or grandparent with type 2 diabetes, or were born to a mother with gestational diabetes mellitus [18], obesity or hypertension. In addition, potential participants were excluded if they had congenital anomalies, developmental delay, chronic diseases, took regular medication or if they were involved in regular programmed physical activity or dietary programs.

Participants were divided into three groups according to birth weight: AGA children, defined on a birth weight between the 10th to 90th percentile for gestational age; SGA children, defined on a birth weight less than or equal to −2 SD, with evidence of catch-up growth [actual height SD score (SDS) within 1.3 SD of the target height SDS] to investigate those with the characteristic SGA growth pattern [19]; and LGA children, defined on a birth weight above the 90th percentile for gestational age. Information regarding birth weight and maternal-perinatal history was obtained by parents and ascertained throughout delivery records and health booklets.

### Assessment of the study population during childhood

Anthropometric measurements and blood samples were taken after complete recovery from the illness for which children were admitted to hospital. All children had a physical examination including anthropometric measurements, blood pressure (BP) and pubertal staging (all participants were prepubertal corresponding to Tanner stage 1). Fasting blood samples were obtained to measure glucose, insulin and lipid profile [including total cholesterol, high-density lipoprotein (HDL) cholesterol, low-density lipoprotein (LDL) cholesterol, triglycerides, and triglycerides-to-HDL cholesterol (triglycerides:HDL) ratio]. Fasting glucose and insulin concentrations were used to calculate the homeostasis model assessment of insulin resistance (HOMA-IR).

### Assessment of the study population during adolescence

The whole study cohort was invited for reevaluation at least 2 years from the first visit. Participants had a repeated physical examination including anthropometric measurement, BP and pubertal staging. Fasting blood samples were obtained to assess fasting glucose, insulin and lipid profile. Thus, the anthropometric and laboratory measurements were consistent between the childhood and adolescent assessments. In this second evaluation, all subjects had pubertal characteristics, corresponding to stage ≥3 (3 = mid-puberty, 4 = late puberty, 5 = post-puberty, respectively).

### Anthropometric measurements

Body weight was determined to the nearest 0.1 kg, and height was measured with a Harpenden stadiometer to the nearest 0.1 cm. Body mass index (BMI) was calculated as weight/height^2^ and expressed as kg/m^2^. Subjects with BMI >95th percentile for the age and sex were defined as obese. Height and BMI SDS were calculated based on the age and sex reference values for Italian children using the LMS method [20]. Maternal and paternal heights were recorded for all participants. Target height (cm) was calculated using Tanner's formula: (the sum of both parents' heights−13)/2 for girls and (the sum of both parents' heights+13)/2 for boys, respectively [21].

### Blood pressure

All BP measurements were obtained by the same researcher properly trained in the techniques of BP assessment. Systolic BP and diastolic BP were measured three times at intervals of at least 1 minute from the non-dominant arm, after five minutes of rest in the supine position using the same calibrated and accurately maintained sphygmomanometer, and then averaged [22].

### Biochemical analysis

Plasma glucose level was determined by the glucose oxidase method, and plasma insulin was measured with a 2-site immunoenzymometric assay (AIAPACK IRI; Tosoh, Tokyo, Japan). The limit of detection was 0.5 µU/mL with intraassay and interassay coefficients of variation of <7%. HOMA-IR was calculated with the formula: fasting insulin (μU/mL) x fasting glucose (mmol/L)/22.5 [23].

### Lipid analysis

Serum total cholesterol, HDL cholesterol, and triglycerides concentrations were determined by the calorimetric enzymatic method. LDL cholesterol was calculated according to the Friedewald formula (LDL cholesterol = total cholesterol−HDL cholesterol−triglycerides/5).

### Score of cardio-metabolic risk

The cardio-metabolic risk was assessed on the basis of the following clinical characteristics and biochemical parameters: BMI, systolic BP, HOMA-IR, triglycerides, and triglycerides:HDL ratio. The sex-specific z-score [z  =  (value−mean)/SD] for each component was calculated based on the mean and the SD of the whole study population (AGA, SGA, and LGA). The sex-specific z-score for each variable was then summed to obtain the clustered cardio-metabolic score for each of the three birth weight groups during childhood and adolescence, respectively. The rationale for selecting this cardio-metabolic score and its components was based on previous studies, in which similar risk score and factors were used [24]–[26].

### Statistical analysis

According to birth weight all children were divided into three groups (AGA, SGA, LGA). Only subjects for whom data for both visits (childhood and adolescence) were available were included in the statistical analysis. All values were expressed as means ± SD unless otherwise stated. Variables of interest non-normally distributed were logarithmically transformed for analyses (insulin, HOMA-IR, and triglycerides). Differences in variables among the three groups were analyzed by general linear model with Bonferroni's test for post hoc comparison of means between each pair of groups. Adjustments for important confounders have been made: BP assessments have been adjusted for subject's height, height has been adjusted for target height, anthropometric measurements (height, weight, BMI) and biochemical parameters (lipid profile) prior to and during puberty have been adjusted for sex, and glucose homeostasis (glucose, insulin, HOMA-IR) has been adjusted for BMI SDS and age. Differences in sex ratio were compared by Fisher's exact test. Paired-Samples T test was performed for evaluating intragroup differences for the clustered risk score and its components between childhood and adolescence. A multiple linear regression analysis was performed to evaluate the independent contribution of birth weight on the clustered risk z-scores obtained during childhood and during adolescence. In addition, a multiple linear regression analysis was performed to evaluate the independent contribution of birth weight and the clustered risk z-score obtained during childhood on the clustered risk z-score obtained during adolescence. In the models, data have been adjusted for age and sex (included as a continuous variable and as a categorical variable, respectively). The birth weight categories have been included as predictors in the regression model. As the use of more than two categorical variables as predictors is not allowed in the multiple regression analysis, dummy coding was used to categorize birth weight, and in the models the birth weight categories were included as dummy variables (AGA *versus* SGA, and AGA *versus* LGA, respectively). *P* values <0.05 were considered statistically significant. SPSS program version 16.0 for Windows was used.

For the purpose of the sample estimation, changes in insulin resistance between childhood and adolescence were based on data from the literature [27]. A sample size of 30 for each group would provide a power of roughly 90% to detect a 1.51 difference in HOMA-IR among groups, with a SD of 0.68, and a 5% significance level.

## Results

The whole study population was recalled for a longitudinal evaluation after at least 2 years from the first visit (mean time interval (±SD) 5.0±2.0 yr). Invitation letters together with an information leaflet about the study were send to the study participants. From the initial 115 participants, 90 adolescents (35 AGA, 24 SGA, 31 LGA; girls:boys ratio approximately 2∶1) agreed to participate in this part of the study and were re-assessed. The remaining 25 subjects from the original study population were not recruited either due to lack of interest in the study, and inability to make contact or failing to meet the inclusion criteria (involvement in regular and programmed physical activity and/or dietary intervention). The primary reasons for losing boys to the longitudinal part of the study were involvement in physical activity programs and a follow-up time not long enough to reach adolescence.

### Childhood assessment

Clinical characteristics and laboratory investigations of the study population during childhood are reported in Table 1.

**Table 1:** Longitudinal evaluation of clinical characteristics and biochemical evaluation of the study population during childhood and adolescence.

	CHILDHOOD (*n* = 90)			ADOLESCENCE (*n* = 90)		
	AGA	SGA	LGA	AGA	SGA	LGA
**Clinical characteristics**						
Number	35	24	31	35	24	31
Age (years)	8.7±1.0	8.6±1.4	8.0±1.7	13.5±1.7	13.4±1.9	13.0±2.0
Sex ratio	11M/24F	8M/16F	10M/21F			
Birth weight (g)	3364±283	2514±136**	4141±223**			
Gestational weeks	39.6±1.2	39.4±1.4	39.7±1.4			
Height SDS	0.5±1.1	0.4±1.0	0.7±0.7	0.3±0.9	0.0±0.9	0.5±0.6
BMI SDS	1.35±1.10	1.59±1.06	1.71±1.04	0.82±1.25	1.36±0.68	1.34±1.01
Obese subjects (%)	34	41	45	14	25	25
Pubertal stage (%)	100	100	100	20/34/46	25/33/42	26/29/45
Systolic BP (mmHg)	103±6	102±5	103±8	115±8	117±6	117±7
Diastolic BP (mmHg)	62±5	62±4	63±5	69±4	71±7	71±5
**Lipid profile**						
Total cholesterol (mg/dl)	160.8±17.6	160.9±11.8	166.9±19.3	163.8±22.4	169.3±18.5	176.2±28.8
HDL cholesterol (mg/dl)	56.4±7.0	55.9±7.4	52.2±6.2	53.6±8.1	52.7±7.3	50.4±3.3
LDL cholesterol (mg/dl)	92.0±17.3	90.8±10.5	98.1±20.1	96.8±24.9	96.3±16.7	108.6±27.4
Triglycerides (mg/dl)	71.1±18.6	72.3±28.1	83.1±25.4	83.7±31.4	87.3±17.0	93.9±26.9
Triglycerides:HDL ratio	1.29±0.44	1.33±0.57	1.53±0.50	1.57±0.67	1.62±0.31	1.58±0.38
**Glucose metabolism**						
Fasting glucose (mg/dl)	78.8±6.1	84.5±8.0**	86.6±6.0**	82.9±4.7	86.6±5.1**	86.4±3.2*
Fasting insulin (μU/ml)	7.7±3.2	12.0±4.8**	13.1±5.4**	13.2±5.8	20.3±6.6**	18.9±4.7**
HOMA-IR	1.55±0.72	2.57±1.20**	2.85±1.24**	2.94±1.18	4.59±1.56**	4.04±0.91**

Values are means (± SD). *P<0.05 and **P<0.01, for comparisons to AGA group by *post hoc* analysis.

Pubertal stage is presented as % in phase 1 during childhood, and as % in phase 3, 4 and 5, respectively, during adolescence.

AGA =  Appropriate for Gestational Age; SGA =  Small for Gestational Age; LGA =  Large for Gestational Age; M =  Male; F =  Female; SDS =  Standard Deviation Score; BP =  Blood Pressure; HDL =  High-Density Lipoprotein; LDL =  Low-Density Lipoprotein; HOMA-IR =  Homeostasis Model Assessment of Insulin Resistance.

The AGA, SGA and LGA groups were similar for gestational weeks, age and sex, whereas a difference was found in birth weight (*P*<0.01). No difference was observed among AGA, SGA and LGA children in height SDS, BMI SDS, BP, and lipid profile.

Fasting insulin and HOMA-IR values were higher in the SGA group compared to AGA subjects (both *P*<0.01). Similarly, LGA children showed higher fasting insulin and HOMA-IR values compared to the AGA group (both *P*<0.01).

### Adolescence assessment

Clinical characteristics and laboratory investigations of the study population during adolescence are reported in Table 1.

The AGA, SGA and LGA groups were similar for age. No difference was found in height SDS, BMI SDS, BP, and lipid profile.

Fasting insulin and HOMA-IR values were higher in SGA adolescents compared to AGA peers (both *P*<0.01). Similarly, LGA adolescents showed higher fasting insulin and HOMA-IR values than the AGA group (both *P*<0.01).

### Intragroup differences in cardio-metabolic markers between childhood and adolescence

An increase in systolic BP and HOMA-IR was found from childhood to adolescence within each birth weight group (all *P*<0.01). A reduction was detected in BMI SDS within LGA and AGA subjects (both *P*<0.05), without considerable difference in the SGA group. Triglycerides remained stable over time in LGA and AGA subjects, while a trend to an increase was observed in the SGA group (*P* = 0.058). No intragroup difference was found in triglycerides:HDL ratio.

### Cardio-metabolic risk score from childhood to adolescence

**Figure 1:**
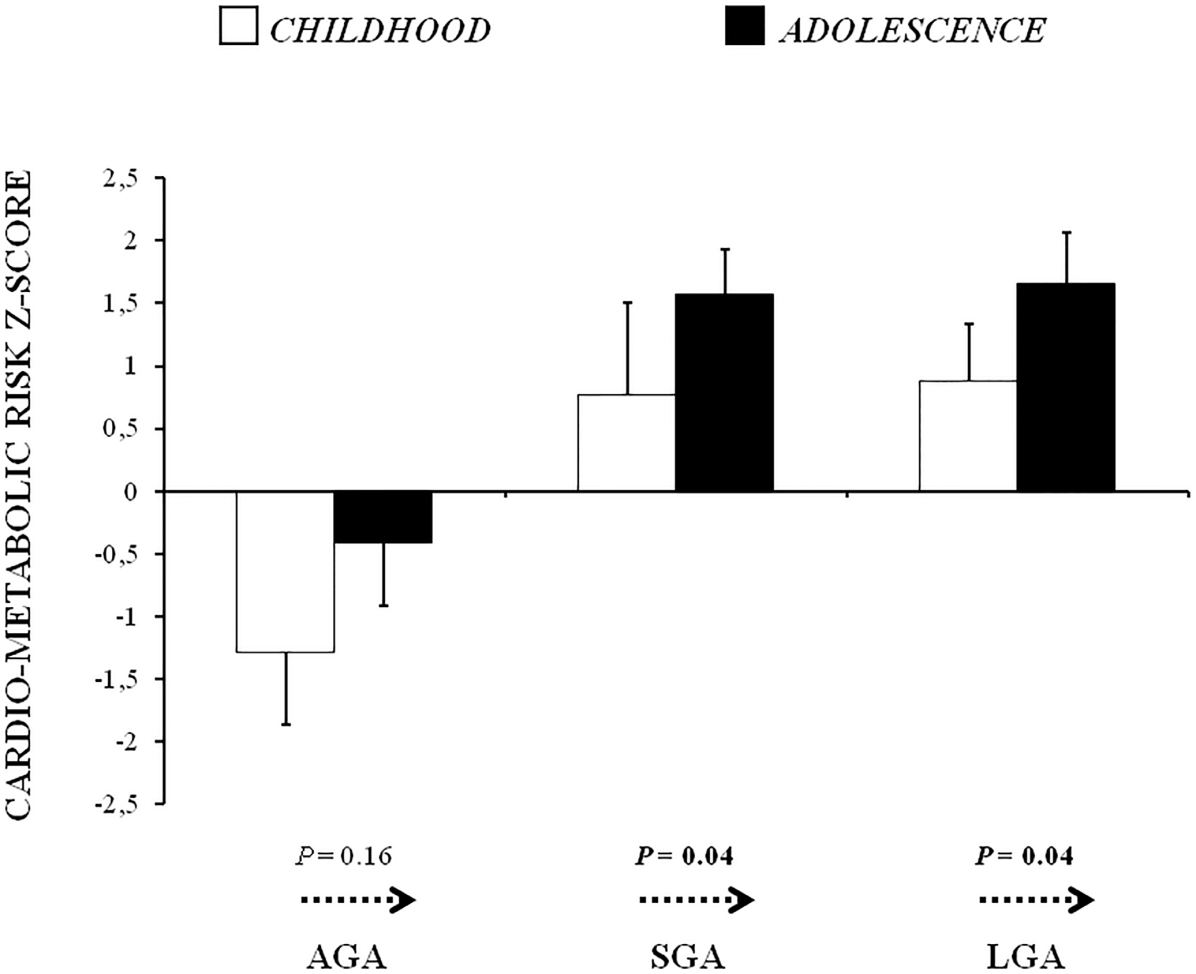
Cardio-metabolic risk z-scores of AGA, SGA and LGA subjects during childhood and adolescence. Values are means (± SE). *P* values by Paired-Samples T test for temporal change (arrows) in the cardio-metabolic risk score from childhood to adolescence within each group. AGA =  Appropriate for Gestational Age; SGA =  Small for Gestational Age; LGA =  Large for Gestational Age.

The clustered risk z-scores of AGA, SGA and LGA subjects during childhood and adolescence are shown in Figure 1.

The clustered risk scores were higher in SGA and LGA children than those born AGA (both *P*<0.05). During adolescence, both SGA and LGA subjects showed higher cardio-metabolic risk scores than AGA peers (*P* = 0.01 and *P*<0.01, respectively).

Of note, a progression of the clustered risk score from childhood to adolescence was observed within the SGA group (P<0.05) and within the LGA group (P<0.05), while no difference was detected in the AGA group.

### Associations between birth weight and the clustered risk z-scores

A multiple linear regression analysis was performed to evaluate the potential associations between birth weight categories and the clustered z-scores during childhood and during adolescence (Table 2). A positive and significant association was found between SGA and LGA categories and the clustered risk z-scores during childhood and adolescence.

**Table 2:** Association between birth weight categories and cardio-metabolic risk z-scores during childhood and adolescence.

	Cardio-metabolic risk z-score			
	*Childhood*		*Adolescence*	
	β Coefficient	*P*	β Coefficient	*P*
AGA *vs* SGA	0.334	0.008	0.344	0.003
AGA *vs* LGA	0.330	0.01	0.409	0.001

Dependent variable: cardio-metabolic risk z-score. *R^2^* during childhood = 0.14. *R^2^* during adolescence = 0.17. Data were adjusted for age and sex.

AGA =  Appropriate for Gestational Age; SGA =  Small for Gestational Age; LGA =  Large for Gestational Age.

In addition, a multiple linear regression analysis was performed to evaluate the potential role of birth weight categories and the childhood risk score as independent predictors for the risk score obtained during adolescence (Table 3). In this model, both the SGA and LGA groups and the childhood clustered z-score were significantly and independently related to the clustered z-score during adolescence.

**Table 3:** Association between birth weight categories, the cardio-metabolic risk z-score during childhood and the cardio-metabolic risk z-score during adolescence.

	Cardio-metabolic risk z-score during adolescence	
	β Coefficient	P
AGA vs SGA	0.328	0.004
AGA vs LGA	0.336	0.005
Cardio-metabolic risk z-score during childhood	0.395	<0.001

Dependent variable: cardio-metabolic risk z-score during adolescence. *R^2^* = 0.37. Data were adjusted for age and sex.

AGA =  Appropriate for Gestational Age; SGA =  Small for Gestational Age; LGA =  Large for Gestational Age.

## Discussion

In this longitudinal study children born SGA and LGA had greater insulin resistance than those born AGA, which was shown to increase from childhood to adolescence. Similarly, the clustered risk score was higher in SGA and LGA children than AGA peers, and these differences among groups increased during adolescence. Notably, a global increase in the risk score was observed within the SGA and the LGA group, suggesting an overtime progression of their cardio-metabolic risk.

A large body of evidence has highlighted that SGA and LGA children are at increased risk of cardio-metabolic diseases at a young age [2], [6], [16]. The first metabolic abnormality seems to be insulin resistance, which can be observed during the first years of life in SGA and LGA children through adolescence [13]–[17], [28]. In a longitudinal analysis performed retrospectively on data collected from the Bogalusa Heart Study, Frontini et al. reported that low birth weight is associated with adverse metabolic outcomes, including altered glucose homeostasis, from childhood to adolescence [29]. In line with these observations, the present study showed greater insulin resistance in the same group of SGA and LGA subjects from childhood to early adolescence. No detectable differences have been found in adiposity, BP and lipid profile among the three birth weight groups. Thus, insulin resistance in itself seems to be a persisting characteristic of both SGA and LGA population. Although the exact mechanisms remain to be determined, prenatal exposure to an adverse metabolic environment and rapid post-natal catch-up growth might play a major role in influencing insulin sensitivity [7], [8], [11], [12].

Interestingly, an emerging concept of clustering cardiovascular and metabolic risk factors has been introduced by several authors to assess subjects at major risk for future diseases [24], [26], [30]. According to this concept, several scores have been proposed combining both cardiovascular and metabolic criteria in adults [24], [30] and children [26], [31], allowing estimation of the individual risk level.

We decided to use a risk score combining both clinical characteristics and biochemical parameters, including adiposity, BP, insulin resistance and lipids, as they can easily be collected in clinical practice and, even more importantly, all of them have been recognized as major predictors of cardiovascular disease [2], [32]–[34]. In addition, in line with previous studies a clustered risk z-score was used to compensate for daily variations in each factor [27], [35], [36]. Of interest, our data suggest a global increase in cardiovascular risk as calculated by the composite risk Z-score from childhood to adolescence in SGA and LGA categories. These findings suggest that early interventions to improve long-term cardiovascular outcomes in those born SGA and LGA may be warranted. Hence, clustering of risk factors in SGA and LGA subjects could be useful in assessing the cardio-metabolic risk and its overtime progression.

Our data support an independent effect of birth weight categories on later metabolic disturbances. Moreover, a rising attractive question is whether childhood cardio-metabolic profile could be associated to the later cardio-metabolic risk score. In this respect, we found a significant association between the childhood score and that one obtained during adolescence, suggesting that the cardio-metabolic profile showed during infancy seems to be predictive of future abnormalities. These findings are in accordance with other studies where infancy and childhood have been recognized as critical time periods for subsequent development of cardiovascular and metabolic alterations [37], [38]. Therefore, childhood may represent an important window of opportunity to optimize risk factors in SGA and LGA populations.

It needs to be acknowledged that, although several scores have been proposed to identify adults and youths at potential high risk for future diseases [24]–[26], [30], defining accurate clusters of risk factors still remains a challenge. Thus, using a composite risk score may represent a limitation in terms of overfitting and validation as risk scores must be validated with actual cardiovascular and diabetes outcomes to find out if the composite score is more predictive than individual components. In addition, we used risk z-scores based on a relatively small cohort and not on population norms.

A further limitation is the exclusion of children following exercise and dietary programs, which may represent an important bias due to the potential missing of subjects with the highest cardiovascular risk. Although this exclusion criterion may limit generalizability of our finding to the wider population, we chose children who did not regularly exercise to increase the ability to detect a difference between groups. In addition, this ensured that physical activity was similar between groups as exercise has insulin sensitizing effects, which may have obscured differences in insulin resistance between birth weight categories.

## Conclusions

SGA and LGA populations are known to have increased cardio-metabolic risk factors in childhood and adulthood. Our study adds to this literature showing that there is progression of these risk factors as children enter early adolescence. Further longer longitudinal studies are needed to elucidate the mechanisms responsible for progression of cardio-metabolic risk factors from infancy to adolescence in SGA and LGA subjects.
